# MYC Protein Expression in Primary Diffuse Large B-Cell Lymphoma of the Central Nervous System

**DOI:** 10.1371/journal.pone.0114398

**Published:** 2014-12-05

**Authors:** Kamraan Z. Gill, Fabio Iwamoto, Ashleigh Allen, Daniela Hoehn, Vundavalli V. Murty, Bachir Alobeid, Govind Bhagat

**Affiliations:** 1 Department of Pathology and Cell Biology, Columbia University Medical Center, New York, NY, 10032, United States of America; 2 Department of Neurology, Columbia University Medical Center, New York, NY, 10032, United States of America; Istituto dei tumori Fondazione Pascale, Italy

## Abstract

Primary diffuse large B-cell lymphoma of the central nervous system (CNS DLBCL) is a rare, aggressive subtype of DLBCL, the biology of which is poorly understood. Recent studies have suggested a prognostic role of MYC protein expression in systemic DLBCL, but little is known about the frequency and significance of MYC protein expression in CNS DLBCL. Hence, we investigated MYC protein expression profiles of CNS DLBCL and assessed the relationship between MYC expression and a variety of histopathologic, immunophenotypic, genetic, and clinical features. Fifty-nine CNS DLBCL diagnosed at our institution over the past 13 years were evaluated. The majority of cases (80%) showed centroblastic morphology, and 12 (20%) displayed a perivascular pattern of infiltration. According to the Hans criteria, 41 (69%) cases had a non-germinal center B-cell and 18 (31%) had a germinal center B-cell cell-of-origin (COO) phenotype. Mean MYC protein expression was 50% (median: 50%, range: 10-80%). Forty-three cases (73%) showed MYC overexpression (≥40%), and 35 (60%) showed MYC/BCL2 coexpression. MYC overexpression was seen in the single case harboring *MYC* translocation and in the cases showing increased copies of *MYC* (27%); however, no significant difference in mean MYC expression was seen between groups harboring or lacking *MYC* aberrations. In our series, age was associated with a significantly increased risk of death, and the perivascular pattern of infiltration was associated with a significantly increased risk of disease progression. Neither MYC expression (with or without BCL2 coexpression) nor other variables, including COO subtype were predictive of clinical outcome. Our findings indicate that the proportion of CNS DLBCL overexpressing MYC is higher compared to systemic DLBCL, and MYC overexpression appears to be independent of genetic *MYC* abnormalities. Thus, MYC expression and other immunophenotypic markers used for prognostication of systemic DLBCL might not apply to CNS DLBCL due to differences in disease biology.

## Introduction

Primary diffuse large B-cell lymphoma of the central nervous system (CNS DLBCL) is a subtype of diffuse large B-cell lymphoma (DLBCL) arising in the brain or an intraocular location. Exclusion criteria for diagnosis according to the 2008 WHO classification include dural involvement, intravascular large B-cell lymphoma, immunodeficiency-associated lymphoma, and secondary lymphoma [1]. CNS DLBCL is rare, accounting for <1% of all non-Hodgkin lymphomas and approximately 3% of all primary brain neoplasms [2]. Compared to systemic DLBCL, CNS DLBCL has a poor prognosis, and it remains unclear whether this reflects a location specific or microenvironmental effect, inherent aggressive biology of the tumor, or a combination of these factors. The cell-of-origin (COO) of CNS DLBCL is thought to be a late germinal center B-cell based on immunophenotypic analysis, gene expression profiling, and molecular characterization of the immunoglobulin genes [3], [4]. The biology of CNS DLBCL is poorly understood due to its rarity and, in part, due to difficulty in obtaining sufficient tumor samples.

*MYC* is a pleiotropic transcription factor that has important roles in diverse cellular processes, ranging from the regulation of cell size, survival, proliferation, metabolism, and signal transduction to control of DNA replication. It is also a potent oncogene, and deregulation of *MYC* expression due to genetic lesions (e.g., translocations, amplifications, and mutations) or other mechanisms is one of the more common alterations in cancer, having been implicated in the pathogenesis of many types of human neoplasms including a variety of solid tumors and lymphoid malignancies [5]. Recent studies have described the utility of cytogenetic and FISH analyses in characterizing *MYC* aberrations, which can aid in distinguishing between Burkitt lymphoma and other types of aggressive B-cell lymphomas [6]. *MYC* rearrangements have been reported in 10–15% of systemic DLBCL, and they have been associated with a poor prognosis either alone or in combination with *BCL2* rearrangements (“double-hit” lymphomas) [7]–[9]. DLBCL displaying high MYC protein expression have also been shown to have an adverse prognosis independent of *MYC* rearrangement and COO subtype [10]–[12]. The incidence of *MYC* rearrangements has been reported to be slightly lower in CNS DLBCL (3–8%), and limited data thus far have not shown any prognostic impact of these rearrangements [13], [14]. *MYC* mutations bearing the molecular hallmarks of aberrant somatic hypermutation have also been detected in CNS DLBCL, but their clinical significance is unknown at present [15]. Increased transcripts of *MYC* and MYC-induced genes involved in cell survival and proliferation were reported by gene expression profiling of CNS DLBCL compared to nodal DLBCL, potentially implicating MYC deregulation in disease pathogenesis [16]. Data regarding MYC protein expression in CNS DLBCL are limited, with disparate frequencies of expression having been reported (0–92%) [13], [17], [18]. Importantly, the association, if any, of MYC expression levels with biological and clinical features has not been established in CNS DLBCL. Thus, we sought to determine MYC protein expression profiles of a large series of CNS DLBCL diagnosed and treated at our institution to investigate the association between MYC protein expression and a variety of histopathologic, immunophenotypic, and clinical features. Cytogenetic and FISH analyses to assess chromosome 8q24 and *MYC* genetic aberrations were performed in a subset of cases.

## Methods

### Ethics statement

This study was performed in accordance with the regulations of the Columbia University Human Research Protection Program and a protocol approved by the Institutional Review Board of Columbia University, New York, USA. All patients provided written informed consent for use of tissue samples for clinical research, and the study was conducted according to the principles expressed in the Declaration of Helsinki.

### Case selection

We searched our departmental database for all cases of DLBCL involving the CNS diagnosed over the past 14 years. The 2008 WHO criteria were used to classify cases as CNS DLBCL after excluding lymphomas associated with immunodeficiency (e.g., HIV infection or post-organ transplantation), those arising in or involving the dura, cases with prior or concurrent low-grade B-cell lymphomas, and secondary CNS involvement by systemic DLBCL [1]. Cases with insufficient material to allow adequate interpretation of immunohistochemical stains were also excluded. Clinical and radiographic data were obtained from the electronic medical record system or from patient charts by the neurooncologist (FI).

### Morphology

H&E-stained sections were used for cytomorphologic evaluation. Patterns of neoplastic cell infiltration were classified as either 1) perivascular (neoplastic lymphocytes localized around blood vessels with only limited parenchymal infiltration) or 2) diffuse (large aggregates or sheet-like infiltrates of neoplastic lymphocytes).

### Immunohistochemistry

The antibodies used included CD10 (monoclonal mouse anti-human antibody, clone 56C6, predilute, Novocastra, Buffalo Grove, IL), BCL6 (monoclonal mouse anti-human antibody, clone LN22, predilute, Novocastra, Buffalo Grove, IL), MUM1/IRF4 (monoclonal rabbit anti-human antibody, clone MRQ-43, predilute, Dako, Carpinteria, CA), BCL2 (monoclonal mouse anti-human antibody, clone bcl-2/100/D5, predilute, Novocastra, Buffalo Grove, IL), MIB-1 (monoclonal rabbit anti-human antibody, clone 30-9, predilute, DAKO, Carpinteria, CA), and C-MYC (monoclonal mouse anti-human antibody, clone 9E10, dilution 1∶100, Epitomics Inc., Burlingame, CA). Staining was performed with an automated staining machine (Universal Staining System, DAKO) after moist heat induced antigen retrieval, and Envision Plus (DAKO) was used for visualization with Diaminobenzidine as the chromogen, according to standard protocols. The percentage of tumor cells expressing the aforementioned markers was recorded in deciles by two pathologists (KG and GB), and the COO subtype was determined using the Hans criteria [19].

### Cytogenetic analysis

G-band karyotype analysis was performed on metaphase preparations of fresh tumor specimens, where available, after unstimulated overnight cultures, and karyotypes were described according to the International System for Human Cytogenetic Nomenclature [20]. FISH was performed on formalin fixed paraffin embedded tissue according to standard protocols using LSI c-MYC break-apart probes (Abbott Molecular, Des Moines, IL, USA). Fluorescence signals were captured after counterstaining with 4′,6-diamidino-2-phenylindole (DAPI) using the Cytovision Imaging system attached to a Nikon Eclipse 600 microscope (Applied Imaging, Santa Clara, CA, USA). The range of normal variation (95% confidence interval) of the *c-MYC* probe was 1.0+0.6% cells showing *MYC* breaks in formalin-fixed, paraffin-embedded control (tonsil) tissue. Hybridization signals were interpreted by two observers (KG and VM) in all cases.

### Statistical analysis

Chi-square, Fisher's exact, and Student's t-tests were used to compare histopathologic, immunophenotypic, and clinical variables as appropriate. Pearson's correlation was used to compare percentage MYC expression and the Ki-67 proliferation index. Univariate and multivariate logistic regression models were used to analyze risk of disease progression and death associated with pathological and clinical variables. Progression-free (PFS) and overall survival (OS) were evaluated using Kaplan Meier analysis with a log rank comparison. Survival analyses were restricted to the patients receiving treatment and follow up at our institution. A p-value <0.05 was considered significant for all analyses. Statistical analysis was performed as applicable with the following software: SPSS version 20 (IBM, Armonk, NY), Excel (Microsoft, Redmond, WA), and Prism (Graph Pad, La Jolla, CA).

## Results

### Clinical characteristics

Fifty-nine cases of CNS DLBCL met our selection criteria. The median age of patients at diagnosis was 65 years (range: 37–87 years), and 24 (41%) were male. Pertinent clinical features are listed in Table 1, and the complete data set is provided in Table S1. On imaging, 50 (88%) cases were supratentorial, and 24 (41%) showed involvement of deep-brain structures (i.e., brain stem, cerebellum, basal ganglia, corpus callosum, or the ventricles). Thirty of 57 (53%) patients with available data had multiple tumors. LDH levels at diagnosis were elevated in 16 of 23 (70%) patients with available data. The median Karnofsky Performance Status (KPS) was 70% (range: 20–90%), and 34 of 58 (59%) patients with available data had ECOG scores ≥2. CSF protein levels were unavailable for the vast majority of cases. Forty-three of 48 (90%) patients with available treatment data received therapy. Twenty-seven (63%) patients received methotrexate monotherapy, while 13 (30%) received rituximab and 7 (16%) received whole brain radiation therapy (WBRT) in addition to chemotherapy. The untreated patients died before initiation of treatment, refused treatment, or were not treated due to their overall medical condition. Details of the therapeutic modalities are listed in Table 1.

**Table 1:** Clinical Characteristics of CNS DLBCL.

Variable	Result
Age at diagnosis (years)	Median: 66, range: 37–87
Sex (males)	24/59 (41%)
KPS	Median: 70%, range: 20–90%
ECOG	Mean: 2, cases ≥2: 34/58 (59%)
LDH*	Elevated: 16/23 (70%), range: 224-1931 units/L
**Tumor location:**	
Supratentorial	52/59 (88%)
Infratentorial	7/59 (12%)
**Number of tumor masses:**	
Single	27/57 (47%)
Multiple	30/57 (53%)
**Deep brain structure involvement:**	
Yes	24/59 (41%)
No	35/59 (59%)
**Therapy:**	
MTX	26/43 (60%)
MTX, WBRT	1/43 (2%)
MTX, rituximab	1/43 (2%)
R-MVP	2/43 (5%)
R-MVP, WBRT	1/43 (2%)
R-MVP, cytarabine	4/43 (9%)
R-MVP, cytarabine, WBRT	2/43 (5%)
MTX, rituximab, temozolamide	1/43 (2%)
MTX, rituximab, temozolamide, WBRT	1/43 (2%)
MTX, rituximab, procarbazine, WBRT	1/43 (2%)
MVP, cytarabine, temozolamide	1/43 (2%)
MTX, thiotepa	1/43 (2%)
MTX, taxol, WBRT	1/43 (2%)
No therapy	5/48 (10%)

Abbreviations: MXT, methotrexate. WBRT, whole brain radiation therapy. R-MVP, rituximab, methotrexate, procarbazine, and vincristine.

*Normal range of LDH: 115–221 units/L.

Of the treated patients with follow-up information, median OS was 14.1 months and median PFS was 6.0 months. Lymphoma was the cause of death for all patients followed until death. Twenty-one patients (36%) were lost to follow up and 10 patients (17%) were still alive at the time of data collection. The follow-up time of the latter ranged from <1 month to 128 months (mean: 22 months, median: 9 months).

### Morphology

**Figure 1:**
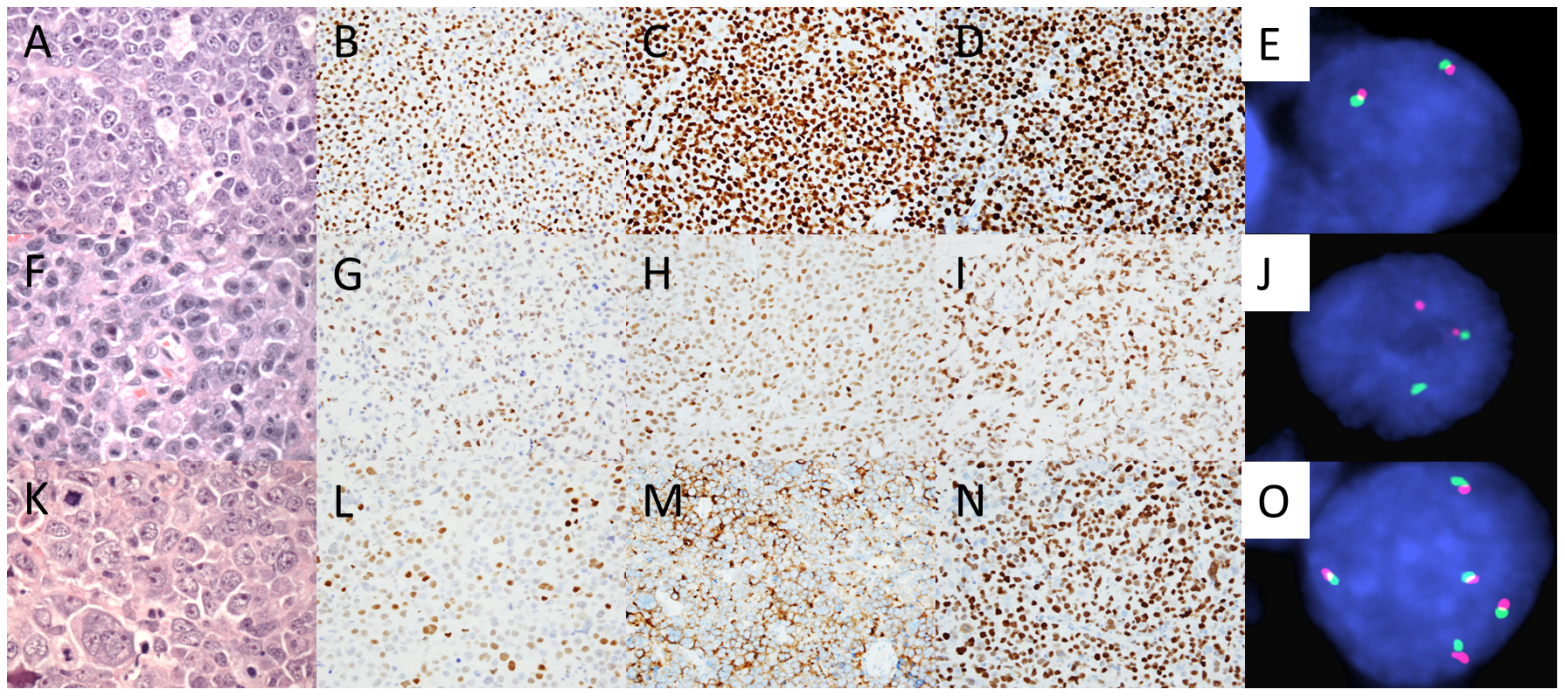
Representative examples of CNS DLBCL harboring or lacking chromosome 8/*MYC* aberrations. H&E-stained section of a case exhibiting morphologic features intermediate between DLBCL and Burkitt lymphoma (A), 80% MYC expression by the neoplastic cells (B), non-GCB COO subtype - BCL6+ (C) and MUM1/IRF4+ (D), and displaying two normal *MYC* loci by FISH (two fused signals) (E). H&E-stained section of a case showing immunoblastic morphology (F), 50% MYC expression by the neoplastic cells (G), non-GCB COO subtype - BCL6+ (H) and MUM1/IRF4+ (I), and displaying *MYC* rearrangement by FISH (one split signal, red and green, and one fused signal) (J). H&E-stained section of a DLBCL showing anaplastic morphology (K), 60% MYC expression by the neoplastic cells (L), GCB COO subtype - CD10+ (M) and BCL6+ (N), and increased copies of *MYC* by FISH (5 fused signals) (O). Photomicrographs of all H&E-stained sections were taken at 400x magnification and all others at 40x magnification.

**Figure 2:**
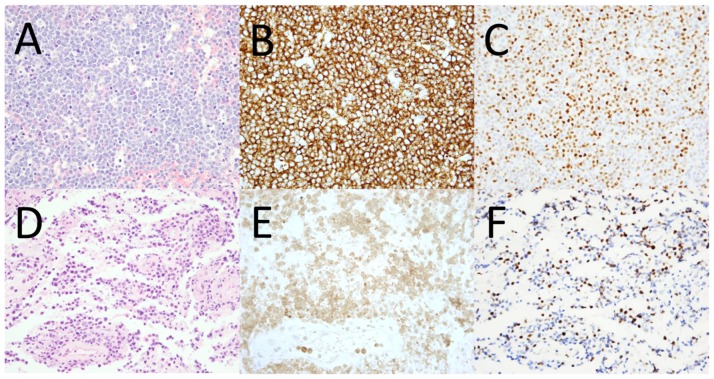
Representative examples of patterns of CNS DLBCL infiltration and MYC protein expression. H&E-stained section of a CNS DLBCL exhibiting a diffuse pattern of infiltration (A), CD20 expression (B), and MYC expression by 80% of neoplastic cells (C). H&E-stained section of a CNS DLBCL exhibiting a perivascular pattern of infiltration (D), CD20 expression (E), and MYC expression by 30% of neoplastic cells (F). All photomicrographs were taken at 40x magnification.

Forty-seven (80%) cases were classified as centroblastic, two of which exhibited signet ring cell features; 2 (3%) were immunoblastic; 1 (2%) was anaplastic; four (7%) exhibited plasmacytoid morphology; and 5 (8%) had morphologic features intermediate between DLBCL and Burkitt lymphoma (representative cases are illustrated in Figure 1). Twelve cases (20%) showed a perivascular pattern of infiltration, and the remainder showed a diffuse pattern (representative examples are shown in Figure 2).

### Immunohistochemistry

Using the Hans criteria, 41 (69%) cases had a non-GCB COO phenotype (CD10-/BCL6-/MUM1/IRF4+, n = 4; CD10-/BCL6+/MUM1/IRF4+, n = 37) and 18 (31%) had a GCB COO phenotype (CD10+/BCL6+/MUM1/IRF4-, n = 4; CD10-/BCL6+/MUM1/IRF4-, n = 6; and CD10+/BCL6+/MUM1/IRF4+, n = 8). Of the cases with morphologic features intermediate between DLBCL and Burkitt lymphoma, four had a non-GCB and one had a GCB COO phenotype. The median Ki-67 proliferation index was 70% (mean: 68%, range: 20–100%), and the median MYC expression was 50% (mean: 50%, range: 10–80%) (representative examples are shown in Figures 1 and 2). No significant difference in mean MYC expression was noted between cases manifesting non-GCB and GCB COO phenotypes (52% vs. 46%, respectively, p = 0.14). There was no significant difference in MYC expression between cases exhibiting perivascular and diffuse patterns of infiltration. A weak positive correlation was noted between MYC expression and the Ki-67 proliferation index (r = 0.3). Using cutoffs for MYC and BCL2 positivity of ≥40% and ≥70%, respectively (similar to cutoffs used in prior studies of systemic DLBCL [10], [21]), 43 (73%) cases were considered as overexpressing MYC and denoted MYC+, 41 (71%) were considered BCL2+, and 35 (60%) were considered MYC+ and BCL2+ (“double positive”). The proportion of MYC+ cases was not significantly different between the non-GCB and GCB COO subtypes (76% vs. 67%, respectively, p = 0.53). However, the proportion of BCL2+ cases was significantly higher in the non-GCB compared to the GCB COO subtype (83% vs. 41%, respectively, p<0.005), and there was a trend toward a higher proportion of MYC and BCL2 double positive cases in the non-GCB compared to the GCB COO subtype (68% vs. 41%, respectively, p = 0.08).

### Cytogenetic analysis

G-band karyotypes to assess 8q24 aberrations and/or FISH to assess *MYC* aberrations were performed in 31 of 59 cases (53%). Informative results were obtained in 16 of 31 cases (52%). Karyotypes were obtained in 8 of 12 (67%) cases, and successful FISH analyses were obtained in 11 of 24 (46%) cases; 3 cases had both a successful karyotype and FISH analysis. Of the cases with karyotypes, all except one showed complex karyotypes. Deletion of 6q was the most frequent recurrent abnormality, observed in three (38%) cases. Other recurrent changes, each observed in two cases, were dup(1q), add(14)(q32), add(15)(p11.2), +3, +7, +13, and +21. No case exhibited 8q24 rearrangement by karyotype. Of the 11 cases with informative FISH analyses, one (9%) showed *MYC* rearrangement and three (27%) showed increased copies of *MYC* (mean *MYC* copies/cell: 3, 4, and 6) (see Figure 1 for examples). Two of the cases with increased copies of *MYC* had polyploid karyotypes (1 triploid and 1 tetraploid); the third case did not have a successful karyotype to determine the mechanism of *MYC* copy number increase. MYC protein expression was 50% in the case with *MYC* rearrangement and was 60–80% in the cases showing increased copies of *MYC*. Mean MYC protein expression in the group comprising cases with *MYC* rearrangement or increased *MYC* copies was not significantly different from the group lacking these abnormalities (68% vs. 64%, respectively, p>0.1).

### Association of morphologic, phenotypic, and clinical parameters with outcomes

**Figure 3:**
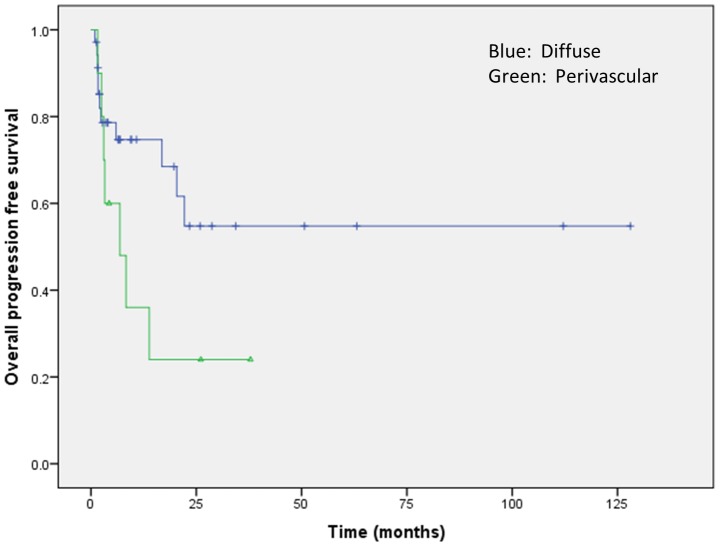
Survival curve showing a trend toward shorter progression-free survival of CNS DLBCL manifesting a perivascular pattern of infiltration.

Analysis of clinical outcomes was restricted to the 43 patients who received therapy at our institution. The perivascular pattern of infiltration was associated with a significantly higher risk of disease progression (odds ratio: 5.09, p<0.05) and a trend toward shorter PFS (p = 0.09; Figure 3). No significant differences in PFS or OS were observed according to the cytomorphologic subtype of DLBCL, individual marker expression (i.e., CD10, BCL6, or MUM1/IRF4), COO subtype, or Ki-67 proliferation index (all p-values >0.1). MYC and/or BCL2 expression were analyzed for impact on survival using all possible deciles as cutoffs and in subsets of patients receiving rituximab or WBRT, but no significant associations were observed.

Older age was associated with a significantly increased risk of death (odds ratio: 1.09, p<0.005), while rituximab therapy was associated with a significantly decreased risk of death (odds ratio: 0.04, p<0.005). Individuals receiving WBRT showed a trend toward lower risk of disease progression (odds ratio: 0.2, p = 0.06) and decreased risk of death (odds ratio: 0.07, p = 0.07). Of note, significant associations of older age with increased risk of death and rituximab with decreased risk of death persisted when analyzing MYC+ and MYC- cases independently. No significant differences in survival were noted when other individual components of the International Extranodal Lymphoma Study Group scoring system (i.e., involvement of deep brain structures, LDH level, and performance scores) were analyzed [22].

On multivariate analysis, older age showed a trend toward increased risk of death (odds ratio: 1.08, p = 0.06), rituximab retained a significant association with decreased risk of death (odds ratio: 0.06, p = 0.02), and perivascular pattern of infiltration retained a significant association with a higher risk of progression (odds ratio: 0.06, p<0.05). WBRT showed neither significant differences nor trends toward decreased risk of progression or death in multivariate analysis. See Table S2 for detailed results of statistical analyses with associated p values.

## Discussion

We evaluated MYC protein expression in conjunction with a variety of morphologic, immunophenotypic, genetic, and clinical parameters in a large single-center series of CNS DLBCL to determine whether MYC protein expression could serve as a prognostic or theranostic biomarker. Similar to prior reports, our CNS DLBCL predominantly displayed centroblastic morphology and the non-GCB COO phenotype (69%). MYC overexpression (≥40%) was noted in 73% of our cases, which is slightly lower that the frequency recently reported by Brunn et al. (92%) but still higher than that described in systemic DLBCL (approximately 30–50%) [9], [10], [12]. Differences in the frequencies of MYC expression between CNS and systemic DLBCL may be related to the predominance of the non-GCB COO subtype in CNS DLBCL, as MYC expression is higher in the ABC (or non-GC) COO subtype of systemic DLBCL [10], [12]. Importantly, in contrast to recent studies of systemic DLBCL [10]–[12], we did not find any association of MYC expression, MYC/BCL2 coexpression, or COO subtype with adverse clinical features or survival. A decreased risk of death was noted in patients receiving rituximab treatment, and an increased risk of disease progression was observed in cases exhibiting a perivascular pattern of infiltration.

The underlying genetic and molecular basis for MYC protein expression in CNS DLBCL is currently unclear. The reported frequencies of *MYC* rearrangement in CNS DLBCL (3–8% in prior studies and 9% in our study) are slightly lower than those reported in systemic DLBCL (10–15%) [7]–[9], [13], [14]. MYC overexpression in a subset of our cases showing increased copies of *MYC* (27%), due to chromosome 8 polysomy, could suggest a dosage effect of the *MYC* gene as an explanation for MYC overexpression in some instances. Increased copies of chromosome 8 have been shown to correlate with increased MYC transcripts in certain myeloid malignancies, but this relationship has not yet been established in lymphomas [23]. Nonetheless, rearrangements and increased copies of *MYC* do not appear to completely account for the high frequency of MYC overexpression in CNS DLBCL observed by us and others [13].

The impact of *MYC* mutations, occurring as a consequence of aberrant somatic hypermutation, on MYC protein expression in CNS DLBCL, remains unexplored [15]. Exonic, hot spot mutations associated with *MYC* deregulation have not been reported in CNS DLBCL. Thus, it remains unclear whether certain *MYC* mutations could contribute to increased MYC protein expression in CNS DLBCL.

Activation of the NF-κB pathway, either directly or indirectly via activation of the B-cell receptor pathway, could represent an alternative mechanism of *MYC* deregulation in CNS DLBCL, since NF-κB is a known transcriptional activator of *MYC* [3]. In this context, similar to systemic ABC or non-GCB DLBCL and other subtypes of B-cell lymphomas, a substantial number of CNS DLBCL harbor chromosome 6q deletions (up to 71% in prior studies and 38% in our study), which can result in the loss of at least two tumor suppressor genes (*PRDM1* and *TNFAIP3*) that have been shown to deregulate NF-κB activity [14], [24]–[26]. Mutations of genes associated with NF-κB activation (e.g., *PRDM1, TNFAIP3, MYD88, TBL1XR1*) or the B-cell receptor signaling pathway (e.g., *CD79b* and *CARD11*) could also lead to increased MYC expression in a subset of CNS DLBCL [3], [24], [27], [28]. The limited number of cases with cytogenetic analysis in our study and the lack of molecular analysis precluded assessment of this hypothesis. Future studies integrating MYC protein expression with assays of NF-κB pathway activation, in the context of the aforementioned chromosomal and genetic alterations, would be worthwhile in determining their role in MYC overexpression in CNS DLBCL.

Studies over the past couple of years have started to uncover complex genetic and epigenetic mechanisms regulating *MYC* expression, stability, and function in cancer, including lymphomas [29]–[32], and interrogation of these might shed light on the mechanisms underlying MYC overexpression in CNS DLBCL. Rubenstein et al. described increased MYC mRNA expression in CNS DLBCL compared to nodal DLBCL by gene expression profiling (GEP) and quantitative RT-PCR analysis [16]. However, Brunn et al. observed similar MYC mRNA levels as described in systemic DLBCL on reevaluating their previously published GEP data and therefore suggested post-transcriptional or post-translational mechanisms for *MYC* deregulation in CNS DLBCL [4], [13]. Deregulation of MYC expression by alterations of microRNAs is also conceivable. Numerous microRNAs have been shown to positively or negatively modulate MYC expression [33]–[35]. Some MYC-regulated microRNAs (miR-17-5p, miR-20a) have been shown to be upregulated in CNS DLBCL [36]. However, a microRNA mediated pathway of MYC overexpression has not yet been described in CNS DLBCL. Increased proliferation may provide yet another alternative explanation for increased MYC expression in CNS DLBCL, as MYC expression has been shown to be closely linked to the cell cycle and is part of the “proliferation signature” in a variety of cancers [29], [37], [38]. We observed a weak positive correlation between the Ki-67 proliferation index and MYC protein expression, while Brunn et al. observed a stronger correlation [13]. Functional studies are, however, required to validate these findings.

*MYC* rearrangements, either alone or in combination with *BCL2* rearrangements (“double-hit” lymphomas), have been associated with resistance or a poor response to therapy and inferior survival in systemic DLBCL [7]–[9], [39]. High MYC and/or BCL2 protein expression, as assessed by immunohistochemistry, has also been shown to have an adverse prognostic impact in systemic DLBCL, independent of *MYC* rearrangement and COO subtype [10]–[12]. In contrast, *MYC* rearrangements have not thus far been shown to be prognostically relevant in CNS DLBCL [13], [14]. The rarity of *MYC* translocation in our series did not allow for any meaningful analysis, and larger studies with uniformly treated patients will be required to confirm or refute prior observations.

Data regarding MYC protein expression in CNS DLBCL is sparse [13], [17], [18]. Two older and smaller series comprising 14 and 27 CNS DLBCL reported MYC expression in 0% and 50% of cases, respectively, with the latter suggesting an association of MYC expression with adverse prognosis [17], [18]. It should be noted that numerically defined cutoffs of MYC expression or overexpression were not used in these studies. A recent report by Brunn et al. described MYC overexpression (defined as >40% MYC expression) in 92% of their cases; however, no associations with clinical outcomes were reported [13]. The prior discrepant frequencies of MYC expression may be due to differences in reagents, staining techniques, or patient populations studied [17], [18]. We did not observe any associations between MYC expression and morphologic, immunophenotypic, or clinical parameters including survival.

The relatively high frequency of BCL2 expression in our series (71%) is within the range described previously (56–92%) [13], [40], [41]. An inconsistent association of higher BCL2 expression with the non-GCB subtype has been reported in systemic DLBCL, and BCL2 expression has been associated with translocations or amplifications involving chromosome 18q21, the locus of *BCL2* [10], [42]. Weber et al. reported 18q gains in 37% of 19 cases of CNS DLBCL, but we and others did not find +18q to be a significant recurrent abnormality [25], [26], [43]. Thus, at present, the contribution of 18q gains to increased BCL2 expression in CNS DLBCL remains unclear. An association between BCL2 expression and the non-GCB COO subtype was seen in our series, but we did not observe an association of BCL2 expression or MYC/BCL2 coexpression with any adverse clinical features.

Prior studies have reported a higher frequency of the non-GCB subtype in CNS DBLCL (74%–97%) compared to systemic DLBCL (approximately 50%) [10], [40]–[42], [44]–[47]. CNS DBLCL of non-GCB COO subtype accounted for a slightly lower proportion of cases in our series (69%). Differences in antibodies, algorithms or technical factors could possibly account for the different frequencies of COO subtypes between our and prior studies. It is unclear whether CNS DBLCL with the phenotype CD10+/BCL6+/MUM1/IRF4+, comprising 14% of our cases, were classified as the non-GCB COO subtype in any of the previous reports. Enrichment in cases of the non-GCB subtype has been suggested to contribute to the poor prognosis of CNS DLBCL [40]. However, we did not detect any adverse effect of COO subtype on survival, findings in line with studies by Hattab et al. and Raoux et al. [45], [46].

A significantly decreased risk of death was observed in patients treated with rituximab, irrespective of MYC expression levels. This finding further corroborates prior data showing improved survival of patients treated with rituximab in CNS DLBCL [48]–[51]. Although we observed trends toward decreased risk of relapse and death in patients treated with WBRT, no significant difference was noted on multivariate analysis.

A variety of other prognostic biomarkers have previously been evaluated in CNS DLBCL. Among these, a perivascular pattern of tumor cell infiltration has been associated with a poor prognosis [52]. In line with this finding, the perivascular pattern of infiltration was an independent predictor of disease progression in our series. We did not evaluate the presence of reactive perivascular T cells, a feature that has been associated with superior overall survival [52], [53] or the microvessel density, which as assessed by morphology or VEGF and endoglin (CD105) expression has previously shown an inconsistent prognostic association in CNS DLBCL [54]–[56]. A few prior studies reported an association of BCL6 expression with better prognosis; however, we and others did not observe such an association [41], [57]–[62]. Finally, 6q deletions and homozygous 9p deletions have been associated with significantly decreased PFS and OS [14], [24]. Due to the limited number of cases analyzed, a statistically meaningful analysis could not be performed in our series.

The strengths of our study include the relatively large number of cases of CNS DLBCL analyzed for MYC expression, MYC/BCL2 coexpression, and COO subtype. This is the largest series thus far to evaluate MYC expression in CNS DLBCL and the first to assess the impact of MYC/BCL2 coexpression on clinical outcomes. The outcomes and follow-up duration of our study are similar to or higher than prior retrospective studies [45], [46], [63]. Comparisons of survival outcomes between retrospective studies like ours (and many prior ones) and those reporting results of prospective clinical trials, however, are not appropriate as patients are highly selected in the latter. The limitations of our study include incomplete data regarding therapy, clinical follow up, and cytogenetic and molecular analyses to correlate with MYC protein expression. Lastly and importantly, while we make several comparisons of CNS DLBCL with systemic DLBCL, these should be interpreted with caution, as treatment regimens for these two types of lymphoma are different. Further studies comprising larger numbers of cases with complete genetic analyses and clinical follow up would be useful to validate and expand upon our findings and help elucidate prognostically relevant biomarkers.

## Supporting Information

Table S1 **Clinical and pathologic features of CNS DLBCL.**
(XLSX)

Table S2 **Details of statistical analyses.**
(XLSX)
